# Autosomal recessive primary microcephaly (MCPH): clinical manifestations, genetic heterogeneity and mutation continuum

**DOI:** 10.1186/1750-1172-6-39

**Published:** 2011-06-13

**Authors:** Saqib Mahmood, Wasim Ahmad, Muhammad J Hassan

**Affiliations:** 1 Department of Human Genetics and Molecular Biology, University of Health Sciences, Khayaban-e-Jamia Punjab, Lahore, 54600, Pakistan; 2 Department of Biochemistry, Faculty of Biological Sciences, Quaid-i-Azam University, Islamabad, 44000, Pakistan; 3 Department of Biochemistry, University of Health Sciences, Khayaban-e-Jamia Punjab, Lahore, 54600, Pakistan

**Keywords:** Heterogeneity, Mutations, Pakistan, MCPH

## Abstract

Autosomal Recessive Primary Microcephaly (MCPH) is a rare disorder of neurogenic mitosis characterized by reduced head circumference at birth with variable degree of mental retardation. In MCPH patients, brain size reduced to almost one-third of its original volume due to reduced number of generated cerebral cortical neurons during embryonic neurogensis. So far, seven genetic loci (MCPH1-7) for this condition have been mapped with seven corresponding genes (*MCPH1*, *WDR62*, *CDK5RAP2*, *CEP152*, *ASPM*, *CENPJ*, and *STIL*) identified from different world populations. Contribution of *ASPM *and *WDR62 *gene mutations in MCPH World wide is more than 50%. By and large, primary microcephaly patients are phenotypically indistinguishable, however, recent studies in patients with mutations in *MCPH1, WDR62 *and *ASPM *genes showed a broader clinical and/or cellular phenotype. It has been proposed that mutations in MCPH genes can cause the disease phenotype by disturbing: 1) orientation of mitotic spindles, 2) chromosome condensation mechanism during embryonic neurogenesis, 3) DNA damage-response signaling, 4) transcriptional regulations and microtubule dynamics, 5) certain unknown centrosomal mechanisms that control the number of neurons generated by neural precursor cells. Recent discoveries of mammalian models for MCPH have open up horizons for researchers to add more knowledge regarding the etiology and pathophysiology of MCPH. High incidence of MCPH in Pakistani population reflects the most probable involvement of consanguinity. Genetic counseling and clinical management through carrier detection/prenatal diagnosis in MCPH families can help reducing the incidence of this autosomal recessive disorder.

## Introduction

In this article, we have reviewed the clinical and molecular genetics studies of autosomal recessive primary microcephaly (MCPH). This review article has been divided into three main sections. In the first part, we have discussed the clinical manifestations of the disease. In second part, molecular genetics of autosomal recessive primary microcephaly including a comprehensive appraisal of the seven mapped loci (MCPH1 - MCPH7), their corresponding genes, protein products of the genes, their likely role in normal brain development and the details of the mutations reported in these genes, especially with reference to Pakistan, have been reviewed. Lastly, probable involvement of MCPH genes in the evolution of human brain to perform diverse cognitive functions has been apprehended.

## Clinical Manifestations

Autosomal Recessive Primary Microcephaly (MCPH- MIM 251200) is a developmental defect of brain characterized by congenital reduction in head circumference of at least 2 standard deviations (SD) below ethnically matched, age- and sex-related mean and non-progressive mental retardation of variable degree, in absence of any significant neurological deficit [1-8]. Microcephaly in MCPH is primary i.e. it is evident by 32^nd ^week of gestation, is present at birth, and is non-progressive [4,5,9,10]. Head circumference (HC) or occipitofrontal circumference (OFC) is the most common diagnostic tool for MCPH. It has been observed that HC ranges from 2 SD to 11 SD below the mean in MCPH patients with very mild interfamilial difference in degree of microcephaly [8,11-13]. By using the definition of HC 3 SD or below the mean, MCPH patients with normal intelligence are very rare [14,15]. Most of the MCPH patients are easy-to-handle and can acquire several self-help skills. An indistinguishable sloping forehead is present in many cases but not always found to be associated with MCPH [11,16,17]. Seizures and epilepsy have been found in higher frequency in patients with *ASPM *mutations, but are very rarely found in patients affected due to other MCPH genes, and are no more common than in general population [4,8,12,18]. Table 1 gives a description of phenotypes for MCPH patients.

**Table 1:** A depiction of clinical symptoms associated with MCPH

MCPH features associated with Apparent phenotype	MCPH features associated with MRI-based/cellular phenotype
Microcephaly (at least 2 SD below the mean) at birth, mild to severe, non-progressive mental retardation (borderline IQ is possible).	Reduction of cerebral cortical volume (neuronal proliferation defect) and simplification of gyral pattern in most cases
Epilepsy/fits/seizures are very rare but cannot exclude the diagnosis *(ASPM)*	Premature chromosome condensation in some cases with increased prophase-like cells *(MCPH1)*
Sloping forehead is although common but is not always associated	Pachygyria with cortical thickening as well as hypoplasia/agenesis of the corpus callosum (neuronal migration defect) in some cases *(ASPM, WDR62)*
Delay in early motor milestones (Speech delay is common)	In few cases Lissencephaly, schizencephaly, polymicrogyria and, cerebellar hypoplasia have also been observed *(WDR62)*
Some times aggressive but otherwise have a happy effect (easy to handle)	
Short stature in some cases *(MCPH1) *	

### *Do MCPH1*, *WDR62 *and *ASPM *mutations result in different phenotype?

Earlier, based on the findings of several studies by different groups on more than 200 families world-wide, it was believed that specific physical appearance of patients linked to each MCPH locus is indistinguishable [11,12,16,17,19,20]. Also, chromosomal analysis for most of the MCPH patients was reportedly normal [12]. But recent studies, supported by modern neuro-diagnostic modalities like of Computed Tomography (CT) Scan and Magnetic Resonance Imaging (MRI), have significantly increased our knowledge regarding the discernible clinical presentations of MCPH and expanded the definition of MCPH based on phenotype [4,21,22].

For *MCPH1 *related MCPH patients, in addition to relatively small height, high proportion of prophase-like cells due to premature chromosome condensation is reported [23-25]. In case of MCPH2 gene (*WDR62*) mutations, a broad clinical phenotype in MCPH patients has been observed by three independent research groups [21,26,27]. It includes microcephaly, pachygyria with cortical thickening as well as hypoplasia of the corpus callosum. In some patients, presence of lissencephaly, schizencephaly, polymicrogyria and, in one instance cerebellar hypoplasia has been observed. These were previously regarded as distinct entities from MCPH [26,27].

A recent study has clinically evaluated patients with *ASPM *mutations and showed several additional phenotypes. They include border-line microcephaly and intelligence quotient (IQ) scores, mild epilepsy, late-onset seizures, simplified gyral pattern, ventricle enlargement, partial corpus callosum agenesis, and mild cerebellar hypoplasia [8]. In another report, MRI imaging of the brain of MCPH patients with *ASPM *mutations revealed simplified anterior gyral pattern with reduced white matter, but without any evidence for a neuronal migration defect [28,29].

## Molecular Genetics of MCPH

### Incidence of MCPH

As reported in birth defect registers world-wide, the incidence of microcephaly varies from 1.3 to 150 per 100,000 depending upon the type of population and the range of SD used to define microcephaly [30-33]. It has been found that MCPH is more common in Asians and Arabs than in White; a white (non-consanguineous) MCPH incidence is 1 per million as compared to 1/100,000 in consanguineous populations like Pakistan [1,4,34].

### Genes in MCPH

MCPH is thought to be a disorder of neurogenic mitosis due to the fact that most of the genes implicated in the etiology of MCPH are involved somehow in mitosis and are expressed in neuroepithelium during embryonic neurogenesis [3]. Understanding the neuro-developmental etiology leading to MCPH is of high significance for understanding the brain development and mammalian evolution with a massive increase in size of the cerebral cortex in primates. Genetic heterogeneity is well established in MCPH and so far seven loci (MCPH1-MCPH7) have been discovered, named according to their order of discovery [35-42]. Mutations in seven genes with in these loci have been identified (Table 2). The *ASPM *gene at MCPH5 locus and *WDR62 *at MCPH2 locus have been found mutated in more than 55-60% of the MCPH families jointly, while *CDK5RAP2 *gene at MCPH3 locus has least involvement in causing MCPH [3,4,11,43]. Many of these genes have been found mutated in families from Pakistan with *CDK5RAP2 *mutations found in Pakistani families only [43-45]. Model organisms like Drosophila, Mouse and Zebra fish have been extensively used to study the effects of mutations in different human MCPH genes. Table 3 gives a brief description of animal models for MCPH genes.

**Table 2:** A brief description of known MCPH loci/genes so far

Locus	Chromosome	Gene	Cellular location	Proposed Function	References
MCPH1	8p23	*MCPH1 *Microcephalin	Nucleus/Chromatin	DNA Damage repair, chromosome condensation	[39,52,58]
MCPH2	19q13.12- q13.2	*WDR62*	Nucleus, cetrosomal during mitosis	Not yet established, expression pattern resembles with *ASPM*	[21,26,27,40]
MCPH3	9q33.2	*CDK5RAP2*	Centrosome/Midbody	Regulation of microtubule function/Centrosome maturation	[44,62,63]
MCPH4	15q15-q21	*CEP152*	Centrosome	Involved in centriol duplication	[41,46]
MCPH5	1q31.3	*ASPM*	Pericentrosomal/Midbody	Orientaion of mitotic spindles during embryonic neurogenesis	[22,67,71,79]
MCPH6	13q12.12	*CENPJ*	Centrosome/Midbody	Centriole length control/microtubule function	[44,86,87]
MCPH7	1p33	*STIL*	Pericentrosomal	Spindle organization/Cell cycle progression	[42,96-100]

**Table 3:** Tabular representation of MCPH animal models

Human Gene for MCPH	Animal Ortholog/s of Human Gene	Animal Model Phenotype	References
*MCPH1 */Microcephalin	*mcph1*^-/-^(drosophila/mice)	Reduced protein recruitment to centrosomes and formation of abnormal spindles, missegregation of DNA, aberrant cytokinesis, changes in cell-cycle progression and defects in DNA damage repair. Adult brain sizes are reported to be normal in drosophila and infertility has been shown in mice	[1,45,56,59]
*CDK5RAP2*	*Cnn*^-/- ^(drosophila)	Centrosome dysfunction loss of connection between Centrosomes and pericentriolar matrix) Only subtle defects of asymmetric divisions and Brains are reportedly of normal size	[1,45,68]
*ASPM*	*asp*^-/-^(drosophila) *Aspm*^-/-^(mice)	(drosophila) incorrect centrosome as microtubule-organizing center, mitotic spindle positioning defect, cell cleavage plane positioning defect during asymmetric cell division and spermatogenesis defect (mice) defects in meiosis I and II, affecting the coordination of chromosome segregation and spindle positioning, transgene rescues the microcephaly phenotype but does not produce a gain of function. reduce fertility in males and females mice but do not alter copulation frequency.	[1,45,82-84]
*CENPJ*	*SAS-4*^-/-^(drosophila, c. elegans)	(drosophila) loss of centrioles, abnormal spindle formation and DNA segregation defects. Mutant flies develop into morphologically normal adults without cilia or flagella and die shortly after birth (*c*. *elegans*) loss of centrioles, abnormal control of centrosome size (centrosome organization)	[1,45,92-95]
*STIL*	*Sil*^-/-^(mice) *csp*^-/- ^(zebra fish)	(mice) knockout of SIL is embryonic lethal at E10.5. Between E7.5 and E8.5. the knockout embryos are smaller, display pericardial swelling, midline neural tube defects, failure of neural tube closure and holoprosencephaly. Failure in left-right specification. Due to block in Sonic Hedgehog (Shh) signalling, and increased apoptosis	[1,98-100]
		(Zebra fish) embryonic lethal with increased number of mitotic cells with defects including mono-polar spindles, loss of polarity, misaligned chromosomes, and broadened spindle poles.	

A detailed description of the MCPH gene mutations, their most likely functions in causation of the disease and animal model studies of MCPH genes is as follows.

### 1- Microcephalin (*MCPH1*)

*MCPH1*/microcephalin gene (MIM 607117) at chromosome 8p23 contains 14 exons and 835 amino acids; the genomic size is 241905 bp with three isoforms reported so far and 8032 bp Open Reading Frame [25,46]. Microcephalin, the encoded protein is implicated in DNA damage induced cellular responses and chromosome condensation. This protein is considered to have a role in G2/M checkpoint arrest via maintenance of inhibitory phosphorylation of cyclin-dependent kinase 1 [3]. The first mutation in *MCPH1 *gene was found in MCPH families from Pakistan [25]. Later mutation in this gene was reported in a disorder of microcephaly, short stature and misregulated chromosome condensation, known as Premature Chromosome Condensation Syndrome (PCC). It was therefore discovered that both MCPH1 and PCC syndrome are allelic disorders originated from mutations with in the same gene [24]. More recently, Farooq et al, (2010) [47] described the Craniosynostosis-Microcephaly with Chromosomal Breakage (CMCB) and other abnormalities that were caused by a truncating mutation (302C-G) in exon 4 of the *MCPH1 *gene. CMCB is found to be allelic to PCC syndrome and MCPH1.

A missense mutation has been reported in *MCPH1 *gene demonstrating less severe cellular phenotype and mild microcephaly [4,23]. Also, a large deletion mutation encompassing first 6 exons of Microcephalin gene in an Iranian family, showing autosomal recessive mental retardation and mild microcephaly, has been reported [48].

Microcephalin contains three (one N-terminal and two C-terminal) BRCT domains (Carboxy-terminal domain of breast cancer gene BRCA1); ortholog genes with similar domain structures are also found in model organisms such as the mouse, *Drosophila melanogaster *and *Caenorhabditis elegans *[49]. The BRCT domains are evolutionarily conserved phosphor-peptide binding amino acid tandem repeat domains involved in cell cycle control. Expression studies show high levels of Microcephalin expression localized to the developing forebrain and, in particular, to the walls of the lateral ventricles in fetal brain [49,50]. The gene was also shown to be expressed in fetal mouse brain during neurogenesis, with highest expression in the ganglionic eminences and lateral ventricles [25]. However, it is also expressed at similar levels in many other fetal tissues such as liver and kidney and, at lower levels, in other tissues.

Cell cycle checkpoints, the regulatory pathways that govern the order and timings of cell cycle transition, are inter related with DNA damage response signaling pathways as cell cycle has to be arrested until damage is repaired. A role for microcephalin in DNA damage response was confirmed after the demonstration that microcephalin localizes to DNA repair foci and that cells depleted for microcephalin by RNA interference exhibit failure in cell cycle checkpoints [51]. It has been proposed that mutations in microcephalin are a causal link between impaired DNA damage response signaling and microcephaly and that misregulated chromosome condensation in MCPH1 is mediated by condensin II protein [52,53]. Wood et al (2007) [54], demonstrated that microcephalin protein is recruited to double-strand breaks via interaction of its C-terminal BRCT domains with phosphorylated H2AX, a histone variant that marks sites of damaged DNA, further expanding the signal to CHK1 and CHK2 via subsequent phosporylation, leading to the modulation of cell cycle, DNA repair and apoptosis.

Alderton et al (2006) [55] found defective G2/M checkpoint arrest, nuclear fragmentation after DNA damage, and supernumerary mitotic centrosomes in human lymphoblastoid cell lines with different truncating mutations in *MCPH1*. They concluded that MCPH1 has a role in maintaining inhibitory CDK1 phosphorylation, which prevents premature entry into mitosis. Thus, microcephaly occurring in patients with *MCPH1 *mutations may be due to a defective centrosomal function of microcephalin, influencing the number of proliferative cell divisions of neuronal stem cells in neurogenesis.

Brunk et al (2007) [56], and Rickmyre et al (2007) [57], have used Drosophila to evaluate the effects of mcph1 deficiency. The cellular phenotypes include uncoordinated centrosome and nuclear cycles, slowed mitotic entry, premature chromosome condensation, mitotic entry with un-replicated DNA, genomic instability, premature separation and centrosome detachment. They also noted chk2-mediated mitotic arrest in the Drosophila cells with mcph1 deficiency. Surprisingly, the adult brains were of normal size but have defects in mushroom body structure.

Recently, two research articles claim the success in generation of mouse model for *MCPH1 *related primary microcephaly. Trimborn et al, (2010) [58], reported the first mammalian model with mis-regulated mitotic chromosome condensation due to defective Mcph1 function. Although residual wild type allele could be detected by quantitative real-time PCR, cell cultures generated from mouse tissues bearing the homozygous gene trap mutation display the cellular phenotype of misregulated chromosome condensation that is characteristic for the human disorder; thus confirming defective Mcph1 function due to the gene trap mutation. While surprisingly the DNA damage response (formation of repair foci, chromosomal breakage, and G2/M checkpoint function after irradiation) appears to be largely normal in cell cultures derived from Mcph1^gt/gt ^mice, the overall survival rates of the Mcph1^gt/gt ^animals are significantly reduced compared to wild type and heterozygous mice. Moreover, the animals show no obvious physical phenotype and no reduced fertility.

Liang et al (2010) [59] also described the generation of Mcph1 deficient mouse model. They found that Brit1^-/- ^(double negative mice) and mouse embryonic fibroblasts (MEFs) both were hypersensitive to c-irradiation. MEFs and T-lymphocytes exhibited severe chromatid breaks and reduced Rad51 foci formation after irradiation. Notably and in contrast to the findings of Trimborn et al (2010) [58], Brit1 double negative mice were infertile and meiotic homologous recombination was impaired. They observed that Brit1-deficient spermatocytes exhibited a failure of chromosomal synapsis, and a meiotic arrest was observed at late zygotene of prophase I accompanied by apoptosis.

### 2- *WDR62 *(MCPH2)

The gene lies within the MCPH2 candidate linkage interval at chromosome 19q13.12 with 32 functional exons and genomic size of 50230 bp [26,46]. The gene has two predicted isoforms and the protein consisting of 1523 residues contains 15 WD repeats [46].

In three recently published research articles, authors have found recessive mutations in human WD repeat domain 62 gene (*WDR62*) in families showing linkage at MCPH2 locus. Authors have observed variety of severe cerebral cortical malformations in the affected individuals including microcephaly, pachygyria with cortical thickening as well as hypoplasia of the corpus callosum [21,26,27]. The authors reported that some patients with mutations in *WDR62 *had evidence of additional abnormalities including lissencephaly, schizencephaly, polymicrogyria and, in one instance, cerebellar hypoplasia, all traits traditionally regarded as distinct entities.

Bilguvar et al (2010) [26] have found 5 homozygous *WDR62 *mutations including deletions and premature terminations in patients suffering from microcephaly and some additional phenotypes, belonging to Turkish origin. Nicholas et al (2010) [27] have identified *WDR62 *as the second most common cause of MCPH after finding homozygous missense and frame-shifting mutations in seven MCPH families, including one from Pakistan. Yu et al (2010) [21] also identified six families with syndrome of congenital microcephaly and diverse defects in cerebral cortical architecture. They identified six mutations in *WDR62 *gene by use of targeted high throughput sequence analysis. In mice and humans, WDR62 expression is found in neural progenitors within the ventricular and subventricular zones. It is observed that expression of *WDR62 *in the neocortex is transient; spanning the period of embryonic neurogenesis [21]. According to the observation of authors, WDR62 does not apparently associate with centrosomes and is predominantly nuclear in localization [21]. But other researchers have found the expression pattern of WDR62 identical to that of ASPM; another MCPH protein expressed at and able to focus the spindle poles in neural precursor cells [26]. They have elaborated that in interphase cells, WDR62 had weak, diffuse cytoplasmic expression. During mitosis, WDR62 accumulated strongly at the spindle poles but was not present at the midbody in cytokinesis.

Another research group has shown that WDR62 demonstrates notable cell cycle-dependent localization. They have revealed punctate, perinuclear expression of WDR62 during interphase, suggesting localization to the Golgi apparatus, by immunofluorescence staining of endogenous WDR62 in HeLa cells with an anti-WDR62 antibody. In contrast, in HeLa or HEK cells in M phase, the authors have found the presence of WDR62 protein at the spindle poles, as demonstrated by double labeling with γ-tubulin and dynein [27].

These findings have suggested that mutations in *WDR62 *are involved in the pathogenesis of varying cortical abnormalities that until now have largely been conceptualized to be distinct and these diverse features can have unified underlying causation. Although there were no imaging studies presented in the previous mapping of this locus, recent findings by different groups suggest that *WDR62 *is the MCPH2 gene and have extended the phenotype beyond ordinary microcephaly.

### 3. *CDK5RAP2 *(MCPH3)

Bond et al. (2005) [44] identified mutations in cyclin dependant kinase 5 regulatory associated protein 2 (*CDK5RAP2*) gene at MCPH3 locus (MIM 608202). The gene contains 38 exons, genomic size **is **191290 bp, with 5682 bp ORF and the deduced protein contains 1893 amino acids [44,45,60]. Multiple transcript variants exist for this gene on chromosome 9q33.2, but the full-length nature of only two has been determined.

The CDK5RAP2 protein is predicted to contain one N-terminal spindle associated domain and two potential chromosome segregation ATPase (SMC, structural maintenance of chromosomes) domains, which are known to play a role in the cohesion and condensation of chromosomes during mitosis [3]. CDK5RAP2 protein interacts with the gamma-tubulin ring complex (gTuRC) at its N-terminus, with CDK5 regulatory subunit 1 (CDK5R1) at C-terminal interacting site and also contains several coiled-coil domains [44,46].

Bond et al. (2005) [44] identified a homozygous base substitution in exon 4, 243T→A (S81X) resulting in amino acid change, S81X, in one family from Pakistan. In the other Pakistani family they identified a homozygous mutation in intron 26, IVS26-15A→G creating a new superior splice acceptor site, leading to the addition of four amino acids and a termination codon (E385fsX4). Hassan et al. (2007) [43] described recurrence of a previously known mutation (S81X) in MCPH family from Northern Pakistan and concluded that the exact nomenclature for this mutation was 246T > A (Y82X). They also concluded that recurrence of Y82X mutation in *CDK5RAP2 *gene in a family of Kashmiri origin may indicate confinement of this rare haplotype within Northern Pakistani population.

CDK5RAP2 is a centrosome-based protein, and its mRNA is widely expressed in human and in embryonal mouse tissue [44,61]. Nagase et al (2000) [62], have shown that gene expression of C*dk5rap2 *in murine sections is highest in the brain and the spinal cord, particularly in the neuroepithelium of the frontal cortex early in neurogenesis. The mouse protein cdk5rap2 is localized throughout the pericentriolar material in all stages of cell cycle and associates with the γTuRC (γ- tubulin ring complex) via a short conserved sequence present in several related proteins found in a range of organisms from fungi to mammals [63]. Perturbing human CDK5RAP2 protein function delocalized γ-tubulin from the centrosomes and inhibited centrosomal microtubule nucleation, thus leading to disorganization of interphase microtubule arrays and formation of anastral mitotic spindles [63].

Zhang et al (2009) [64] recently demonstrated that CDK5RAP2 is required for spindle checkpoint regulation. They found that loss of its function leads to chromosome mis-segregation and reduced expression of spindle checkpoint proteins, via interaction with their promoters and transcription regulation in HeLa cells. Lucas and Ruff (2007) [65], have found centrosome dysfunction; centrosome 'rocketing' within the cells due to loss of connection between centrosomes and pericentriolar matrix in human CDK5RAP2 ortholog, 'centrosomin,' deficient Drosophila embryos (*cnn-/-*). Only subtle defects of asymmetric divisions in 'centrosomin,' deficient Drosophila embryos (*cnn-/-*) were observed, and brains were reportedly of normal size. Previously, Terada et al (2003) [66] provided the evidence about the interaction of Drosophila orhtholog of CDK5RAP2, centrosomin (*cnn*), with γ-tubulin ring complexes within the centrosome, which is responsible for the production of microtubules. Thus, CDK5RAP2 may affect neurogenic mitosis by affecting the accessibility of the microtubules that are required to assemble the mitotic spindles.

### 4. *CEP152 *(MCPH4)

Guernsey et al (2010) [67] have recently discovered both homozygous and heterozygous mutations in *CEP152 *gene located with in MCPH4 linkage interval at chromosome 15q21.1 in two Canadian families with MCPH using SNP-based genome scan. Human *CEP152 *gene, which is the putative mammalian ortholog of Drosophila *asterless (asl)*, spans 72835 bp with protein product of 152 kDa and 1710 amino acids for largest transcript variant [46].

Previously, Blachon et al (2008) [68] have demonstrated that Asl (ortholog of *CEP152 *in Drosophila) is a conserved gene required for ciliogenesis; is involved in the initiation of centriole duplication and that it is dispensable for pericentriolar material (PCM) recruitment in meiosis in MC centrioles (Mother Centrioles). They were successful to show that asl is essential for centriole formation in brain cells and spermatocytes. The generated mechanosensory mutant D, mecD, (a mutant allele of *asl*) exhibited all of the characteristics of a ciliary defect with a severe phenotype of uncoordination and nonmotile sperm tails and also confirmed the absence of cilia by serial-section electron microscopy (EM) of the sensory neurons and the sperm tails.

However, in mice, Guernsey et al (2010) [67] were able to generate a cDNA clone product of the correct size spanning exons 21-24 of the mouse Cep152 ortholog by using RNA extracted from mouse brain tissue; cDNA could be readily detected in stage 12.5 and 14.5 embryonic brains. They have also used tools of bioinformatics to elaborate the pathogenic effect of homozygous mutation in *CEP152 *gene (Q265P) in two MCPH4 linked families [67].

### 5. *ASPM *(MCPH5)

Bond et al. (2002) [22] identified mutations in *ASPM *(Abnormal Spindle-like Microcephaly-Associated), a human ortholog of the *Drosophila melanogaster *'abnormal spindle' gene (asp), which is essential for normal mitotic spindle function in embryonic neuroblast at MCPH5 locus. The gene spans 62567 bp with 10906 bp ORF [46]. The *ASPM *gene contains 28 exons and deduced protein contains 3477 amino acids, comprising of a putative microtubule-binding domain at its, N-terminal with ciliary function [69-71], a calponin-homology domain (CH), 81 isoleucine-glutamine (IQ) motifs which act as calmodulin-binding domains [22,69,70] and a carboxy terminal region with no identifiable domains [70,72]. The number of *ASPM *IQ domains is constant among diverse mammals, with the exception of some rodents and, are thought to be involved in increased cerebral cortical size in mammalian evolution [71,72].

Mutations in *ASPM *gene (discovered so far) are protein-truncating; they are distributed throughout the gene without any observed association of phenotypic severity with mutation position or type and are the most common cause of MCPH World-wide [11,16,17,19,22,73,74]. These mutations include single base-pair changes, deletions, duplications and intronic variants. Also, a significant number of the *ASPM *mutations have been in compound-heterozygous form [9,16].

ASPM is expressed in the cerebral cortical ventricular zone and proliferative zones of the medial and lateral ganglionic eminences during neurogenesis [22]. Several alternatively spliced variants, encoding putative ASPM isoforms with different numbers of IQ motifs have been identified suggesting the existence of isoforms with potentially different functions [71]. Fish et al (2006) [75], revealed that Aspm (orthologue for mouse protein) protein localizes to spindle poles, and its siRNA mediated knockdown decreases proliferative symmetric divisions in developing neocortex of mouse. They have shown that ASPM maintains symmetrical cell divisions and is down-regulated with the switch from proliferative to neurogenic divisions. Also, it has been found that N- and C-termini of ASPM differentially localize within mitotic cells to either spindle poles or to midbodies, respectively [76]. In general, ASPM expression has been linked to proliferation and is highest in progenitor cells. Mouse Aspm is down regulated upon neurosphere differentiation, and knockdown of Aspm reduced both the self-renewal capacity and proliferation of neurospheres upon re-culture [1]. ASPM might also contribute to adult neurogenesis, as stimulating proliferation of neural precursors in adult rat hippocampus induced Aspm expression [77].

In some recent studies, expression of ASPM in cancer cells has been investigated. Aspm has been found down-regulated in ionizing-radiation treated cells [78], and its down-regulation has shown to decrease Brca1 protein expression as well [79]. In another study, ASPM mRNA was found over-expressed in transformed human cell lines and tumors, and its increased expression was positively associated with proliferation of glioblastoma cells [80].

ASPM plays a role in mitotic spindle function including orientation of cleavage plane. The spindle apparatus dictates the plane of cell cleavage, which is critical in the choice between symmetric or asymmetric division [4,81,82]. Drosophila *asp *gene mutants were the first to be called as mini-brain. Gonzalez et al (1990) [83], described mitotic arrest in these flies with centrosome did not functioning correctly as microtubule organizing center. Drosophila *asp *gene is known to be essential for both the organization and binding together of microtubules at the spindle poles, the formation of the central mitotic spindle and to focus the poles of the mitotic spindles during mitosis and meiosis [81,82]. The mitotic spindle positioning was defective, along with the defects in cell cleavage plane positioning during asymmetric cell division and spermatogenesis.

Pulvers et al (2010) [84] generated two mutant mouse lines from gene trap cells to study the function of Aspm in the development of the cerebral cortex and elsewhere. They discovered that the mutations in Aspm reduce brain size in mice, similar in nature to, albeit with less severity than, human primary microcephaly. They concluded that possible origins of this difference in severity may lie in the difference in brain size itself. They also found that human ASPM transgene rescues the microcephaly phenotype but does not produce a gain of function in mice. They have demonstrated that mutations in Aspm reduce fertility in males and females mice but do not alter copulation frequency.

Given the known functions of Drosophila and mouse orthologs of ASPM, it is, therefore, proposed that mutations of *ASPM *might reduce brain size by influencing mitotic spindle orientation, and reduce neural cell number through an effect on the ratio of asymmetric to symmetric cell divisions [1,82].

### 6. *CENPJ/CPAP *(MCPH6)

Bond et al. (2005) [44] identified two mutations in Centromere-associated Protein J (*CENPJ*) gene in one Brazilian and two Pakistani families within a previously identified locus on human chromosome 13q12.2 [41]. The *CENPJ *gene (MIM 609279) spans 40672 bp with 5187 bp ORF [46]. It has 17 exons and deduced 1,338-amino acid protein contains 5 coiled-coil domains, the most C-terminal of which includes a leucine zipper motif, several potential protein phosphorylation sites and a C-terminal domain containing 21 nonamer G-box repeats [44,85]. It also contains a 112- amino acid long microtubule destabilizing motif PN2-3 [86] and two C-terminal 14-3-3 binding sites [87]. Wide expression of *CENPJ ***is **found with the highest expression in brain and spinal cord; primary expression has been localized to the neuroepithelium of the frontal cortex early in the neurogenesis [44].

Four mutations have been reported in this gene so far. Initially two mutations were reported in three families [44], including two families from Northern Pakistan having one mutation and other from Brazil with second mutation. Later, in a Pakistani family from Pashto language group, a deletion of four consecutive nucleotides (TCAG) was identified in exon 11 of *CENPJ *gene, leading to frameshift and premature termination codon 19 bp downstream in the same exon, predicted to add six amino acids downstream of the mutation [88]. More recently, a novel splicing mutation in *CENPJ *gene [89] has been observed in Seckel syndrome patients.

CENPJ is localized at the centrosome during mitosis and it is concentrated at the mitotic spindle poles during prometaphase and metaphase [44,86]. Recently, Cho et al, (2006) [90], have observed that depletion of CENPJ disrupts centrosome integrity, and cells lacking CPAP arrest in mitosis with multipolar spindles. *In vitro *evidence suggests that CENPJ might be able to inhibit microtubule nucleation and microtubules depolymerization [87].

It has been found that CENPJ depletion by the use of siRNA lead to an increase of multiple spindle poles, mitosis arrest and apoptosis. CENPJ interacts with the N-terminal domain of the p65 subunit (RelA) of NF-kappaB, a transcription factor important for various cellular events such as inflammation, immune response, proliferation and apoptosis [91].

Loss of the CENPJ orthologue dsas-4 in Drosophila leads to the loss of centrioles; although knockout flies survive until adulthood, their coordination is poor and viability is also low [92]. Mutant flies developed into morphologically normal adults without cilia or flagella and died shortly early in life [93]. In *C*. *elegans*, sas-4 is a centriolar protein that controls centrosome size (centrosomal organization) [94]. The role of sas-4 in centrosome duplication was further underlined by FRAP (fluorescence recovery after photo bleaching) studies demonstrating that sas-4 was recruited to the centrosome once per cell cycle, at the onset of the organelle duplication cycle in the S phase [95]. CENPJ is therefore a crucial regulator of centriole length during centriole biogenesis, possibly functioning in the correct recruitment of centriolar microtubules.

### 7- *STIL */ *SIL *(MCPH7)

Seventh locus for primary microcephaly (MCPH7, MIM 612703) was mapped by Kumar et al, 2009 [42] in families with primary microcephaly to a region of 8.39 Mb on chromosome 1p33-p32.3. Sequence analysis of *STIL *(SCL/TAL1-Interrupting Locus) gene that codes for a pericentriolar and centrosomal protein revealed 3 different homozygous protein truncating mutations in affected members of 5 Indian families linked to MCPH7. The *STIL *gene spans 63018 bp with 5225 bp ORF [45]. It contains 20 exons and 1287 residues in full-length protein. STIL is a cytosolic protein of 150 kDa whose function is not fully understood and which lacks homology to any known protein families or motifs [45]. The human full length 1287 amino acid protein contains a putative nuclear localization signal and a C-terminal domain similar to that of TGF-beta [96].

STIL is expressed throughout the cytosol with increased expression in the perinuclear region that likely plays a role in mitotic entry (cell cycle progression G2-M), apoptosis control and centrosome function [42]. Karkera et al (2002) [96], found that the human STIL protein contains a putative nuclear localization signal and a cysteine-terminal domain similar to the C-terminal domain of TGF-beta. It was found by Aplan et al (1990) [97], that genomic rearrangements, containing the *STIL *gene, have been associated with T-cell acute lymphoblastic leukemia (ALL). STIL is phosphorylated in mitosis and then interacts with PIN1 (peptidyl prolyl isomerase) which in turn regulates a subset of mitotic phospoproteins [98]. *STIL *is an early response gene that is ubiquitously expressed in proliferating cells and during early embryonic development [98,99].

Data obtained from zebrafish indicate that stil may not only play a role in centrosome function/duplication, but also in organizing the mitotic spindle. Loss-of-function mutation in zebrafish sil has an embryonic lethal defect [98]. While homozygous sil knockout mice (Sil^-/-^) display several developmental abnormalities around embryonal day (E) 7.5-8.5 and die after E10.5 [100]. They described the phenotype of mutant mice showing reduced size, limited developmental progress, midline neural tube defects, abnormal left-right development, increased rates of apoptosis, decreased rates of proliferation, abnormal expression of several important genes including **"**lefty 2**" **and **"**nodal**"**. Also the mutants were found to be embryonal lethal.

### Mutation spectrum of MCPH Genes in Pakistani Population

Pakistani population constitutes of at least 18 ethnic groups and speaks more than 60 languages [101,102]. Mostly, it belongs to Indo-European speakers from India, although Hazaras, Pathans, Balti, Brusho and Balouch language groups are found to carry West-Asian, Arabian, and European ancestry [101,103,104]. Due to high rate of consanguinity (~61%) and large family size [34], Pakistani population has proved to be rich for genetic linkage studies.

Northern Pakistan is a wide geographical region that harbors people from different language groups e.g. Kashmiri, Shina, Balti, Pashto, Hindko etc. All these groups tend to marry within their close relatives. As a large number of Pakistani MCPH families belong to this region, there is an ample need to detect localization of MCPH gene mutations in specific language groups within this area. This will certainly help in prenatal diagnosis of MCPH and will enhance our knowledge about the dispersion of MCPH alleles in different language groups within Pakistani population.

A total of about 95 families have been reported with mutations in any of MCPH genes from Pakistan, most of them belonging to Northern Pakistan. Except one recently discovered locus from India (MCPH7), all other MCPH loci have been reported in Pakistani families (Tables 4, 5 and 6).

**Table 4:** An account of Pakistani families linked to different MCPH loci and a comparison with other populations

Locus/Gene	**No**. of Pakistani Families linked *	**No**. **of Mutations Reported in Pakistani Families **	**No**. of Families linked worldwide*
**MCPH1 *(Microcephalin) ***	2 (1.39%)	2	8 Iranian families, 1 Caucasian family
**MCPH2 *(WDR62) ***	2 (1.39%)	1	3 Iranian families, 11 Turkish families, 1 Mexican family, 1 Arab family,
**MCPH3 *(CDK5RAP2) ***	3 (2.09%)	2	No family reported outside Pakistan
**MCPH4 *(CEP152) ***	2 (1.39%)	0	3 Canadian families and 1 Arab Family
			
**MCPH5 *(ASPM) ***	83 (58.04%)	79	13 Iranian Families, 12 Arab families, 8 Indian families, about 22 European families, 5 african families, and many sporadic patients
**MCPH6 *(CENPJ) ***	3 (2.09%)	3	5 Iranian Families, 1 Brazilian Family
**MCPH7 *(STIL) ***	0 (0%)	0	3 Indian Families, 2 Iranian Families
	48 Pakistani families (33.56%),		
**Families Not Linked to any locus **	About 120 families from different World Populations have reported to be unlinked to known MCPH loci including 81 from Iran		
			
**Total Families Screened so far**	143 Pakistani families and about 200 families with MCPH World wide have been screened so far		

*- Number of families has been calculated from published data. Research groups working on genetics of MCPH may have more families in their labs.

**Table 5:** *ASPM *gene mutations in MCPH families from Pakistan

Mutation	Language Group	State of Mutation	**Present in No**. **of families from Pakistan**	Reference
349C-T	Northern Pakistani	homozygous	One	[22]
719delCT	Northern Pakistani	homozygous	One	[74]
1258delTCTCAAT	Northern Pakistani	homozygous	Two	[22,74]
9190C-T	Northern Pakistani	homozygous	One	[22]
2936+5G-T (intronic)	Northern Pakistani	homozygous	One	[74]
1990C-T	Northern Pakistani	homozygous	One	[74]
8508delGA	Sindi, Northern Pakistani	homozygous	Four	[16,17,19,74]
3082G-A	Northern Pakistani	homozygous	One	[74]
4581delA	Northern Pakistani	homozygous	One	[74]
4795C-T	Northern Pakistani	homozygous	One	[74]
7895C-T	Northern Pakistani	homozygous	One	[74]
9159delA	Northern Pakistani	homozygous	Three	[22,74,105]
9557C-G	Hindko	homozygous	Three	[11,74]
9984+1G-T (intronic)	Northern Pakistani	homozygous	One	[74]
3663delG	Northern Pakistani	homozygous	Two	[74]
1002delA	Pakistani	homozygous	One	[16]
2938C-T	Pakistani	homozygous	One	[16]
3055C-T	Pakistani	homozygous	One	[16]
7894C-T	Pakistani/heterozygous	Homozygous	Two	[16]
3477delCGCTA	Pakistani	Homozygous	One	[16]
6732delA	Pakistani	Homozygous	One	[16]
8668C-T	Pakistani	Homozygous	One	[16]
3978G-A	Pashto....	Heterozygous/homozygous	Eighteen	[11,16,17,105]
9319C-T	Pakistani	heterozygous	One	[16]
9492T-G	Pakistani	homozygous	Three	[16,105]
9595A-T	Pakistani	homozygous	One	[16]
9677insG	Pakistani	homozygous	One	[16]
9697C-T	Pakistani	homozygous	One	[16]
1260delTCAAGTC	Saraiki	homozygous	Two	[11,105]
3188T-G	Pakistani	homozygous	One	[19]
4855delTA	Pakistani	homozygous	One	[19]
5136C-A	Sindhi	homozygous	One	[17]
5149delA	Saraiki	homozygous	One	[17]
6335delAT	Pakistani	homozygous	One	[24]
7761T-G	Pakistani	homozygous	Two	[19,22]
7782delGA	Pakistani	homozygous	One	[19]
8378delT	Pakistani	homozygous	More than one	[19]
9118insCATT	Punjabi	homozygous	One	[11]
9238A-T	Punjabi	homozygous	Two	[11,19]
9681delA	Pakistani	homozygous	One	[19]
9730C-T	Kashmiri	homozygous	One	[17]
9745delCT	Pakistani	homozygous	One	[19]
9789T-A	Pakistani	homozygous	One	[19]
10059C-A	Saraiki	homozygous	One	[17]
9539A-C	Sindi	homozygous	One	[11]
2101C-T	Pakistani	homozygous	One	[105]
6686delGAAA	Pakistani	homozygous	Two	[8,105]
77delG	Pakistani	homozygous	Two	[8,105]

**Table 6:** *MCPH1*, *WDR62, CDK5RAP2*, and *CENPJ *mutations in MCPH families from Pakistan

Gene Symbol	Mutation	Language Group	State of Mutation	**Present in No**. **of families from Pakistan**	Reference
*MCPH1*	74C-G	Northern Pakistani	Homozygous	Two	[25]
*WDR62*	3232G-A	Sindhi	Homozygous	One	[27]
	246T-A	Kashmiri	Homozygous	Two	[43,44]
*CDK5RAP2*	IVS26-15A-G	Northern Pakistani	Homozygous	One	[44]
	17delC	Northern Pakistani	Homozygous	Two	[44]
*CENPJ*	3243delTCAG	Pushto	Homozygous	One	[88]

*ASPM *gene mutations have been found in 83 families with 2 families having compound heterozygous mutations. Language group is not mentioned for a large number of these families (Northern Pakistan is not a single language group). Two mutations 3978G-A and 8508delGA were most common, segregating in 18 and 4 families respectively (Table 5). The mutation 3978G-A has segregated mostly in Pashto language group. Although, Muhammad et al, (2009) [16] and Kousar et al, (2010) [105] have reported this mutation in families from Pakistan, but not clearly mentioned the language group.

*CDK5RAP2 *gene mutations have been found in three Pakistani families so far, and they are exclusive to Pakistan. One mutation (IVS26-15A-G) was observed in one family while other mutation (Y82X) was present in two families. All these families belong to Northern Pakistan but for only one family, language group has been mentioned.

Two mutations have been found in *CENPJ *gene segregating in three Pakistani families. One mutation 17delC was present in affected individuals from two families belonging to Northern Pakistan. The second mutation (3243delTCAGa) was found segregated in a Pakistani family from Pashto language group.

In one Pakistani family linked to MCPH2 locus Nicholas et al, 2010 [27] have detected a missense mutation c.3232G-A (Ala1078Thr) in exon 27 of the *WDR62 *gene (Table 6).This family belongs to Sindhi language group of southern Pakistan. Previously the locus was reported in Pakistani families only but the mutations in *WDR62 *gene have been found in microcephaly patients from other countries as well [21,26,27].

## Evolution of MCPH Genes and Mammalian Brain Size

MCPH significantly affects the ratio of brain size to body size. Brain size of the affected individuals is comparable to that of early hominids, chimpanzee or gorilla [22,25]. Almost all genes involve in MCPH seem to play a role in mitosis of neuronal precursors. The acceleration of protein evolution has been observed to be most prominent in the lineage leading from ancestral primates to human, therefore, it is concluded that the phenotypic evolution of the human nervous system has a salient molecular correlate, i.e., accelerated evolution of the underlying genes, particularly those linked to nervous system development [106,107]. Many investigators tried to find out evidence for adaptive changes in these genes and also studied the correlation of these changes with change in relative brain size during evolution and cognitive abilities that evolved us as human.

Genetic variants of *ASPM *and *MCPH1 *genes in human have been shown to arise nearly about 5,800 years and 36,000 years ago and have since swept to high frequency under strong positive selection [108-110]. Identification of these remarkably young-aged, positively selected variants suggested that the human brain is still undergoing rapid adaptive evolution. However, it has been suggested that certain developed models of human history including both population growth and spatial structure could generate the observed pattern of *ASPM *and *MCPH1 *haplotypes without selection [111]. Later, it was proposed that these variations are not unusual, do not support selection and ongoing adaptive evolution of *ASPM *and *MCPH1 *is not explained by increased intelligence [112-114]. Molecular evolutionary studies on *CDK5RAP2 *and *CENPJ *also revealed accelerated rates of non-synonymous substitutions within respective functional domains of the genes, in lineage leading to *Homo sapiens *that are likely the result of positive selection [60]. More recently, it has been observed that a common SNP of *MCPH1 *(previously not described) is associated with cranial volume variation in Chinese population without any detectable selection signal, suggesting that the brain volume variation in human populations is likely neutral or under very weak selection in recent human history [115].

The claim that distribution of *ASPM-D *allele and *MCPH-D *allele is the result of positive selection has been challenged by several groups, but arguably remains the best explanation. Also, it has been observed by researchers that the predicted ASPM proteins encode systematically larger numbers of repeated 'IQ' domains between flies, mice, and humans, with the predominant difference between Aspm and ASPM being a single large insertion coding for IQ domains [4,75]. But on the other hand, the expansion of IQ repeats has been observed in other non-primates mammalians [116], and raises the question of the physiological process which really benefits from the selection pressure.

It is important to mention that the derived haplogroups of *ASPM *and *MCPH1 *were apparently not found to be involved in variations in brain size [117], intelligence [112], head circumference, general mental ability, social intelligence [113,114] or the incidence of schizophrenia [118]. It has been proposed by Dediu and Ladd (2007) [119] that effects of derived haplogroups of *MCPH1 *and *ASPM *are involved in subtle differences in the organization of the cerebral cortex, with cognitive consequences including linguistic biases in the processing and acquisition of linguistic tone.

More recently, Montgomery et al (2011) [120] have analyzed the molecular evolution of four genes associated with microcephaly (*ASPM, CDK5RAP2, CENPJ, MCPH1*) across 21 species representing all major clades of anthropoid primates and have found that contrary to prevailing assumptions, positive selection was not limited to or intensified along the lineage leading to humans. Out of four genes tested, they found positive relationships only between *CDK5RAP2 *and *ASPM *and neonatal brain mass and somewhat weaker relationships between these genes and adult brain size. The stronger association of *ASPM *and *CDK5RAP2 *evolution with neonatal brain size than with adult brain size is consistent with these loci having a direct effect on prenatal neuronal proliferation.

The results shown by Montgomery et al (2011) [120] have suggested that primate brain size may have at least a partially conserved genetic basis, and have contradicted previous studies that linked adaptive evolution of *ASPM *to changes in relative cortex size. Their findings about pervasive positive selection in coding regions of two widely expressed loci in relation to a complex, quantitative developmental phenotype have provided a notable counterexample to the commonly asserted hypothesis that cis-regulatory regions play a dominant role in phenotypic evolution.

## Conclusion

One of the most obvious structural characteristics of the human brain, when compared with that of other mammals, is its large size. Cognitive abilities decrease with a significant decrease in brain size and most striking example of it is Autosomal Recessive Primary Microcephaly (MCPH), in which brain size reduces to one third of its original volume with a significant cognitive decline. The MCPH proteins are involved in different molecular pathways including DNA damage response signaling, cell division, proper spindle orientation, microtubule dynamics, cell cycle regulation etc. Recent functional studies confirm the presence of MCPH proteins at centrosome for at least part of cell cycle. Therefore, one may conclude that centrosome is the final integration point for many regulatory pathways affecting prenatal neurogenesis in mammals. The *ASPM *gene at MCPH5 locus and *WDR62 *at MCPH2 locus are the most common cause of MCPH respectively. Recent successful generation of mammalian models for MCPH has open up horizons for researchers to add more knowledge regarding the etiology and pathophysiology of MCPH. Better genotyping, neuro-imaging and neuro-physiological testing, along with constitution of genetically homogeneous groups of patients, would help in establishing exact genotype-phenotype correlation. Mutation screening in MCPH families from Pakistan along with the information about ethnic origin/language group is need of the time, because this population is the most affected group. It will help in genetic counseling and prenatal diagnosis for MCPH in Pakistan, and thus in turn may help reducing the incidence of MCPH from this highly consanguineous population.

## Competing interests

The authors declare that they have no competing interests.

## Authors' contributions

All authors contributed equally in this work in terms of literature search, systematization and writing. All authors read and approve the final submitted version of this manuscript.

## References

[B1] Thornton GK Woods CG Primary microcephaly: do all roads lead to Rome? Trends Genet 2009 25 501 510 10.1016/j.tig.2009.09.011 19850369 PMC2816178

[B2] Mochida GH Genetics and biology of microcephaly and lissencephaly Semin Pediatr Neurol 2009 16 120 126 10.1016/j.spen.2009.07.001 19778709 PMC3565221

[B3] Cox J Jackson AP Bond J Woods CG What primary microcephaly can tell us about brain growth? Trends Mol Med 2006 12 358 366 10.1016/j.molmed.2006.06.006 16829198

[B4] Woods CG Bond J Enard W Autosomal recessive primary microcephaly (MCPH): a review of clinical, molecular, and evolutionary findings Am J Hum Genet 2005 76 717 728 10.1086/429930 15806441 PMC1199363

[B5] Mochida GH Walsh CA Genetic basis of developmental malformations of the cerebral cortex Arch Neurol 2004 61 637 640 10.1001/archneur.61.5.637 15148137

[B6] Mochida GH Walsh CA Molecular genetics of human microcephaly Curr Opin Neurol 2001 14 151 156 10.1097/00019052-200104000-00003 11262728

[B7] McCreary BD Rossiter JP Robertson DM Recessive (true) microcephaly: a case report with neuropathological observations J Intellect Disabil Res 1996 40 66 70 10.1111/j.1365-2788.1996.tb00604.x 8930059

[B8] Passemard S Titomanlio L Elmaleh M Afenjar A Alessandri JL Andria G de Villemeur TB Boespflug-Tanguy O Burglen L Del Giudice E Guimiot F Hyon C Isidor B Mégarbané A Moog U Odent S Hernandez K Pouvreau N Scala I Schaer M Gressens P Gerard B Verloes A Expanding the clinical and neuroradiologic phenotype of primary microcephaly due to *ASPM *mutations Neurology 2009 73 962 969 10.1212/WNL.0b013e3181b8799a 19770472

[B9] Saadi A Borck G Boddaert N Chekkour MC Imessaoudene B Munnich A Colleaux L Chaouch M Compound heterozygous *ASPM *mutations associated with microcephaly and simplified cortical gyration in a consanguineous Algerian family Eur J Med Genet 2009 52 180 184 10.1016/j.ejmg.2009.03.013 19332161

[B10] Desir J Abramowicz M Tunca Y Novel mutations in prenatal diagnosis of primary microcephaly Prenat Diagn 2006 26 989 993 17029297 10.1002/pd.1536

[B11] Gul A Hassan MJ Mahmood S Chen W Rahmani S Naseer MI Dellefave L Muhammad N Rafiq MA Ansar M Chishti MS Ali G Siddique T Ahmad W Genetic studies of autosomal recessive primary microcephaly in 33 Pakistani families: Novel sequence variants in *ASPM *gene Neurogenetics 2006 7 105 110 10.1007/s10048-006-0042-4 16673149

[B12] Roberts E Hampshire DJ Pattison L Springell K Jafri H Corry P Mannon J Rashid Y Crow Y Bond J Woods CG Autosomal recessive primary microcephaly: an analysis of locus heterogeneity and phenotypic variation J Med Genet 2002 39 718 721 10.1136/jmg.39.10.718 12362027 PMC1734986

[B13] Nellhaus G Head circumference from birth to eighteen years: practical composite of international and interracial graphs Pediatrics 1968 4 106 114 5635472

[B14] Abuelo D Microcephaly syndromes Semin Pediatr Neurol 2007 14 118 127 10.1016/j.spen.2007.07.003 17980308

[B15] Teebi AS Al-Awadi SA White AG Autosomal recessive nonsyndromal microcephaly with normal intelligence Am J Med Genet A 1987 26 355 359 10.1002/ajmg.1320260214 3812587

[B16] Muhammad F Mahmood Baig S Hansen L Sajid Hussain M Anjum Inayat I Aslam M Anver Qureshi J Toilat M Kirst E Wajid M Nürnberg P Eiberg H Tommerup N Kjaer KW Compound heterozygous *ASPM *mutations in Pakistani MCPH families Am J Med Genet A 2009 149A 926 930 10.1002/ajmg.a.32749 19353628

[B17] Gul A Tariq M Khan MN Hassan MJ Ali G Ahmad W Novel protein-truncating mutations in the *ASPM *gene in families with autosomal recessive primary microcephaly J Neurogenet 2007 21 153 163 10.1080/01677060701508594 17849285

[B18] Shen J Eyaid W Mochida GH Al-Moayyad F Bodell A Woods CG Walsh CA *ASPM *mutations identified in patients with primary microcephaly and seizures J Med Genet 2005 42 725 729 10.1136/jmg.2004.027706 16141009 PMC1736131

[B19] Nicholas AK Swanson EA Cox JJ Karbani G Malik S Springell K Hampshire D Ahmed M Bond J Di Benedetto D Fichera M Romano C Dobyns WB Woods CG The molecular landscape of *ASPM *mutations in primary microcephaly J Med Genet 2009 46 249 253 10.1136/jmg.2008.062380 19028728 PMC2658750

[B20] Kumar A Blanton SH Babu M Markandaya M Girimaji SC Genetic analysis of primary microcephaly in Indian families: novel *ASPM *mutations Clin Genet 2004 66 341 348 10.1111/j.1399-0004.2004.00304.x 15355437

[B21] Yu TW Mochida GH Tischfield DJ Sgaier SK Flores-Sarnat L Sergi CM Topçu M McDonald MT Barry BJ Felie JM Sunu C Dobyns WB Folkerth RD Barkovich AJ Walsh CA Mutations in *WDR62*, encoding a centrosome-associated protein, cause microcephaly with simplified gyri and abnormal cortical architecture Nat Genet 2010 42 1015 1020 10.1038/ng.683 20890278 PMC2969850

[B22] Bond J Roberts E Mochida GH Hampshire DJ Scott S Askham JM Springell K Mahadevan M Crow YJ Walsh CA Woods CG *ASPM *is a major determinant of cerebral cortical size Nat Genet 2002 32 316 320 10.1038/ng995 12355089

[B23] Trimborn M Richter R Sternberg N Gavvovidis I Schindler D Jackson AP Prott EC Sperling K Gillessen-Kaesbach G Neitzel H The first missense alteration in the MCPH1 gene causes autosomal recessive microcephaly with an extremely mild cellular and clinical phenotype Hum Mutat 2005 26 496 16211557 10.1002/humu.9382

[B24] Trimborn M Bell SM Felix C Rashid Y Jafri H Griffiths PD Neumann LM Krebs A Reis A Sperling K Neitzel H Jackson AP Mutations in microcephalin cause aberrant regulation of chromosome condensation Am J Hum Genet 2004 75 261 266 10.1086/422855 15199523 PMC1216060

[B25] Jackson AP Eastwood H Bell SM Adu J Toomes C Carr IM Roberts E Hampshire DJ Crow YJ Mighell AJ Karbani G Jafri H Rashid Y Mueller RF Markham AF Woods CG Identification of microcephalin, a protein implicated in determining the size of the human brain Am J Hum Genet 2002 71 136 142 10.1086/341283 12046007 PMC419993

[B26] Bilgüvar K Oztürk AK Louvi A Kwan KY Choi M Tatli B Yalnizoğlu D Tüysüz B Cağlayan AO Gökben S Kaymakçalan H Barak T Bakircioğlu M Yasuno K Ho W Sanders S Zhu Y Yilmaz S Dinçer A Johnson MH Bronen RA Koçer N Per H Mane S Pamir MN Yalçinkaya C Kumandaş S Topçu M Ozmen M Sestan N Lifton RP State MW Günel M Whole-exome sequencing identifies recessive *WDR62 *mutations in severe brain malformations Nature 2010 467 207 210 10.1038/nature09327 20729831 PMC3129007

[B27] Nicholas AK Khurshid M Désir J Carvalho OP Cox JJ Thornton G Kausar R Ansar M Ahmad W Verloes A Passemard S Misson JP Lindsay S Gergely F Dobyns WB Roberts E Abramowicz M Woods CG *WDR62 *is associated with the spindle pole and is mutated in human microcephaly Nat Genet 2010 42 1010 1014 10.1038/ng.682 20890279 PMC5605390

[B28] Desir J Cassart M David P Van Bogaert P Abramowicz M Primary microcephaly with *ASPM *mutation shows simplified cortical gyration with antero-posterior gradient pre- and post-natally Am J Med Genet A 2008 146A 1439 1443 10.1002/ajmg.a.32312 18452193

[B29] Vermeulen RJ Wilke M Horber V Krägeloh-Mann I Microcephaly with simplified gyral pattern: MRI classification Neurology 2010 74 386 391 10.1212/WNL.0b013e3181ce5d82 20124203

[B30] International Clearinghouse for Birth Defects Surveillance and Research, 2006 report http://www.icbdsr.org/

[B31] Tolmie JL McNay M Stephenson JB Doyle D Connor JM Microcephaly: genetic counselling and antenatal diagnosis after the birth of an affected child Am J Med Genet 1987 27 583 594 10.1002/ajmg.1320270311 3307411

[B32] Van den Bosch J Microcephaly in the Netherlands: a clinical and genetical study Ann Hum Genet 1959 23 91 116 13637554 10.1111/j.1469-1809.1958.tb01455.x

[B33] Komai T Kishimoto K Ozaki Y Genetic study of microcephaly based on Japanese material Am J Hum Genet 1955 7 51 65 14361394 PMC1716566

[B34] Hussain R Bittles AH The prevalence and demographic characteristics of consanguineous marriages in Pakistan J Biosoc Sci 1998 30 261 275 10.1017/S0021932098002612 9746828

[B35] Jackson AP McHale DP Campbell DA Jafri H Rashid Y Mannan J Karbani G Corry P Levene MI Mueller RF Markham AF Lench NJ Woods CG Primary autosomal recessive microcephaly (MCPH1) maps to chromosome 8p22-pter Am J Hum Genet 1998 63 541 546 10.1086/301966 9683597 PMC1377307

[B36] Roberts E Jackson AP Carradice AC Deeble VJ Mannan J Rashid Y Jafri H McHale DP Markham AF Lench NJ Woods CG The second locus for autosomal recessive primary microcephaly (MCPH2) maps to chromosome 19q131-132 Eur J Hum Genet 1999 7 815 820 10.1038/sj.ejhg.5200385 10573015

[B37] Jamieson CR Govaerts C Abramowicz MJ Primary autosomal recessive microcephaly: homozygosity mapping of MCPH4 to chromosome 15 Am J Hum Genet 1999 65 1465 1469 10.1086/302640 10521316 PMC1288302

[B38] Jamieson CR Fryns JP Jacobs J Matthijs G Abramowicz MJ Primary autosomal recessive microcephaly MCPH5 maps to 1q25-1q32 Am J Hum Genet 2000 67 1575 1577 10.1086/316909 11067780 PMC1287933

[B39] Moynihan L Jackson AP Roberts E Karbani G Lewis I Corry P Turner G Mueller RF Lench NJ Woods CG A third locus for primary autosomal recessive microcephaly maps to chromosome 9q34 Am J Hum Genet 2000 66 724 777 10.1086/302777 10677332 PMC1288125

[B40] Pattison L Crow YJ Deeble VJ Jackson AP Jafri H Rashid Y Roberts E Woods CG A fifth locus for primary autosomal recessive microcephaly maps to chromosome 1q31 Am J Hum Genet 2000 67 1578 1580 10.1086/316910 11078481 PMC1287934

[B41] Leal GF Roberts E Silva EO Costa SM Hampshire DJ Woods CG A novel locus for autosomal recessive primary microcephaly (MCPH6) maps to 13q122 J Med Genet 2003 40 540 542 10.1136/jmg.40.7.540 12843329 PMC1735531

[B42] Kumar A Girimaji SC Duvvari MR Blanton SH Mutations in *STIL*, encoding a pericentriolar and centrosomal protein, cause primary microcephaly Am J Hum Genet 2009 84 286 290 10.1016/j.ajhg.2009.01.017 19215732 PMC2668020

[B43] Hassan MJ Khurshid M Azeem Z John P Ali G Chishti MS Ahmad W Previously described sequence variant in *CDK5RAP2 *gene in a Pakistani family with autosomal recessive primary microcephaly BMC Med Genet 2007 8 58 64 17764569 10.1186/1471-2350-8-58 PMC2072945

[B44] Bond J Roberts E Springell K Lizarraga SB Scott S Higgins J Hampshire DJ Morrison EE Leal GF Silva EO Costa SM Baralle D Raponi M Karbani G Rashid Y Jafri H Bennett C Corry P Walsh CA Woods CG A centrosomal mechanism involving *CDK5RAP2 *and *CENPJ *controls brain size Nat Genet 2005 37 353 355 10.1038/ng1539 15793586

[B45] Kaindl AM Passemard S Kumar P Kraemer N Issa L Zwirner A Gerard B Verloes A Mani S Gressens P Many roads lead to primary autosomal recessive microcephaly Prog Neurobiol 2010 90 363 383 10.1016/j.pneurobio.2009.11.002 19931588

[B46] Human Genome Browser website, 2010 http://genome.ucsc.edu/cgi-bin/hgGateway

[B47] Farooq M Baig S Tommerup N Kjaer KW Craniosynostosis-microcephaly with chromosomal breakage and other abnormalities is caused by a truncating MCPH1 mutation and is allelic to premature chromosomal condensation syndrome and primary autosomal recessive microcephaly type 1 Am J Med Genet A 2010 152A 495 497 10.1002/ajmg.a.33234 20101680

[B48] Garshasbi M Motazacker MM Kahrizi K Behjati F Abedini SS Nieh SE Firouzabadi SG Becker C Rüschendorf F Nürnberg P Tzschach A Vazifehmand R Erdogan F Ullmann R Lenzner S Kuss AW Ropers HH Najmabadi H SNP array-based homozygosity mapping reveals *MCPH1 *deletion in family with autosomal recessive mental retardation and mild microcephaly Hum Genet 2010 118 708 715 10.1007/s00439-005-0104-y 16311745

[B49] Ponting C Jackson AP Evolution of primary microcephaly genes and the enlargement of primate brains Curr Opin Genet Dev 2005 15 241 248 10.1016/j.gde.2005.04.009 15917198

[B50] Xu X Lee J Stern DF Microcephalin is a DNA damage response protein involved in regulation of CHK1 and BRCA1 J Biol Chem 2004 279 34091 34094 10.1074/jbc.C400139200 15220350

[B51] Neitzel H Neumann LM Schindler D Wirges A Tönnies H Trimborn M Krebsova A Richter R Sperling K Premature chromosome condensation in humans associated with microcephaly and mental retardation: a novel autosomal recessive condition Am J Hum Genet 2002 70 1015 1022 10.1086/339518 11857108 PMC379095

[B52] O'Driscoll M Jackson AP Jeggo PA Microcephalin: a causal link between impaired damage response signalling and microcephaly Cell Cycle 2006 5 2339 2344 10.4161/cc.5.20.3358 17102619

[B53] Trimborn M Schindler D Neitzel H Hirano T Misregulated chromosome condensation in MCPH1 primary microcephaly is mediated by condensin II Cell Cycle 2006 5 322 326 10.4161/cc.5.3.2412 16434882

[B54] Wood JL Singh N Mer G Chen J MCPH1 functions in an H2AX-dependent but MDC1-independent pathway in response to DNA damage J Biol Chem 2007 282 35416 35423 10.1074/jbc.M705245200 17925396 PMC2128040

[B55] Alderton GK Galbiati L Griffith E Surinya KH Neitzel H Jackson AP Jeggo PA O'Driscoll M Regulation of mitotic entry by microcephalin and its overlap with ATR signalling Nat Cell Biol 2006 8 725 733 10.1038/ncb1431 16783362

[B56] Brunk K Vernay B Griffith E Reynolds NL Strutt D Ingham PW Jackson AP Microcephalin coordinates mitosis in the syncytial Drosophila embryo J Cell Sci 2007 120 3578 3588 10.1242/jcs.014290 17895363

[B57] Rickmyre JL Dasgupta S Ooi DL Keel J Lee E Kirschner MW Waddell S Lee LA The Drosophila homolog of MCPH1, a human microcephaly gene, is required for genomic stability in the early embryo J Cell Sci 2007 120 3565 3577 10.1242/jcs.016626 17895362

[B58] Trimborn M Ghani M Walther DJ Dopatka M Dutrannoy V Busche A Meyer F Nowak S Nowak J Zabel C Klose J Esquitino V Garshasbi M Kuss AW Ropers HH Mueller S Poehlmann C Gavvovidis I Schindler D Sperling K Neitzel H Establishment of a mouse model with misregulated chromosome condensation due to defective Mcph1 function PLoS One 2010 5 2 e9242 10.1371/journal.pone.0009242 20169082 PMC2821930

[B59] Liang Y Gao H Lin SY Peng G Huang X Zhang P Goss JA Brunicardi FC Multani AS Chang S Li K BRIT1/MCPH1 is essential for mitotic and meiotic recombination DNA repair and maintaining genomic stability in mice PLoS Genet 2010 6 1 e1000826 10.1371/journal.pgen.1000826 20107607 PMC2809772

[B60] Evans PD Vallender EJ Lahn BT Molecular evolution of the brain size regulator genes *CDK5RAP2 *and *CENPJ* Gene 2006 375 75 79 16631324 10.1016/j.gene.2006.02.019

[B61] Ching YP Qi Z Wang JH Cloning of three novel neuronal Cdk5 activator binding proteins Gene 2000 242 285 294 10.1016/S0378-1119(99)00499-0 10721722

[B62] Nagase T Kikuno R Nakayama M Hirosawa M Ohara O Prediction of the coding sequences of unidentified human genes. XVIII. The complete sequences of 100 new cDNA clones from brain which code for large proteins in vitro DNA Res 2000 7 273 281 10997877 10.1093/dnares/7.4.271

[B63] Fong KW Choi YK Rattner JB Qi RZ CDK5RAP2 is a pericentriolar protein that functions in centrosomal attachment of the gamma-tubulin ring complex Mol Biol Cell 2008 19 115 125 17959831 10.1091/mbc.E07-04-0371 PMC2174194

[B64] Zhang X Liu D Lv S Wang H Zhong X Liu B Wang B Liao J Li J Pfeifer GP Xu X CDK5RAP2 is required for spindle checkpoint function Cell Cycle 2009 8 1206 1216 10.4161/cc.8.8.8205 19282672 PMC3820842

[B65] Lucas EP Raff JW Maintaining the proper connection between the centrioles and the pericentriolar matrix requires Drosophila centrosomin J Cell Biol 2007 178 725 732 10.1083/jcb.200704081 17709428 PMC2064537

[B66] Terada Y Uetake Y Kuriyama R Interaction of Aurora-A and centrosomin at the microtubule-nucleating site in Drosophila and mammalian cells J Cell Biol 2003 162 757 763 10.1083/jcb.200305048 12939255 PMC2172831

[B67] Guernsey DL Jiang H Hussin J Arnold M Bouyakdan K Perry S Babineau-Sturk T Beis J Dumas N Evans SC Ferguson M Matsuoka M Macgillivray C Nightingale M Patry L Rideout AL Thomas A Orr A Hoffmann I Michaud JL Awadalla P Meek DC Ludman M Samuels ME Mutations in centrosomal protein *CEP152 *in primary microcephaly families linked to MCPH4 Am J Hum Genet 2010 87 40 51 10.1016/j.ajhg.2010.06.003 20598275 PMC2896783

[B68] Blachon S Gopalakrishnan J Omori Y Polyanovsky A Church A Nicastro D Malicki J Avidor-Reiss T Drosophila asterless and vertebrate Cep152 Are orthologs essential for centriole duplication Genetics 2008 180 2081 2094 10.1534/genetics.108.095141 18854586 PMC2600943

[B69] Saunders RD Avides MC Howard T Gonzalez C Glover DM The Drosophila gene abnormal spindle encodes a novel microtubule-associated protein that associates with the polar regions of the mitotic spindle J Cell Biol 1997 137 881 890 10.1083/jcb.137.4.881 9151690 PMC2139842

[B70] Ponting CP A novel domain suggests a ciliary function for *ASPM*, a brain size determining gene Bioinformatics 2006 22 1031 1035 10.1093/bioinformatics/btl022 16443634

[B71] Kouprina N Pavlicek A Collins NK Nakano M Noskov VN Ohzeki J Mochida GH Risinger JI Goldsmith P Gunsior M Solomon G Gersch W Kim JH Barrett JC Walsh CA Jurka J Masumoto H Larionov V The microcephaly *ASPM *gene is expressed in proliferating tissues and encodes for a mitotic spindle protein Hum Mol Genet 2005 14 2155 2165 10.1093/hmg/ddi220 15972725

[B72] Craig R Norbury C The novel murine calmodulin-binding protein Sha1 disrupts mitotic spindle and replication checkpoint functions in fission yeast J Cell Sci 1998 111 3609 3619 9819352 10.1242/jcs.111.24.3609

[B73] Darvish H Esmaeeli-Nieh S Monajemi GB Mohseni M Ghasemi-Firouzabadi S Abedini SS Bahman I Jamali P Azimi S Mojahedi F Dehghan A Shafeghati Y Jankhah A Falah M Soltani Banavandi MJ Ghani-Kakhi M Garshasbi M Rakhshani F Naghavi A Tzschach A Neitzel H Ropers HH Kuss AW Behjati F Kahrizi K Najmabadi H A clinical and molecular genetic study of 112 Iranian families with primary microcephaly J Med Genet 2010 47 823 828 10.1136/jmg.2009.076398 20978018

[B74] Bond J Scott S Hampshire DJ Springell K Corry P Abramowicz MJ Mochida GH Hennekam RC Maher ER Fryns JP Alswaid A Jafri H Rashid Y Mubaidin A Walsh CA Roberts E Woods CG Protein truncating mutations in *ASPM *cause variable reduction in brain size Am J Hum Genet 2003 73 1170 1177 10.1086/379085 14574646 PMC1180496

[B75] Fish JL Kosodo Y Enard W Pääbo S Huttner WB Aspm specifically maintains symmetric proliferative divisions of neuroepithelial cells Proc Natl Acad Sci USA 2006 103 10438 10443 10.1073/pnas.0604066103 16798874 PMC1502476

[B76] Paramasivam M Chang YJ LoTurco JJ ASPM and citron kinase co-localize to the midbody ring during cytokinesis Cell Cycle 2007 6 1605 1612 10.4161/cc.6.13.4356 17534152

[B77] Gurok U Loebbert RW Meyer AH Mueller R Schoemaker H Gross G Behl B Laser capture micro dissection and microarray analysis of dividing neural progenitor cells from the adult rat hippocampus Eur J Neurosci 2007 26 1079 1090 10.1111/j.1460-9568.2007.05734.x 17767487

[B78] Fujimori A Yaoi T Ogi H Wang B Suetomi K Sekine E Yu D Kato T Takahashi S Okayasu R Itoh K Fushiki S Ionizing radiation downregulates ASPM, a gene responsible for microcephaly in humans Biochem Biophys Res Commun 2008 369 953 957 10.1016/j.bbrc.2008.02.149 18331833

[B79] Zhong X Liu L Zhao A Pfeifer GP Xu X The abnormal spindle-like, microcephaly-associated (*ASPM*) gene encodes a centrosomal protein Cell Cycle 2005 4 1227 1229 10.4161/cc.4.9.2029 16123590

[B80] Hagemann C Anacker J Gerngras S Kühnel S Said HM Patel R Kämmerer U Vordermark D Roosen K Vince GH Expression analysis of the autosomal recessive primary microcephaly genes *MCPH1 *(microcephalin) and MCPH5 (*ASPM*, abnormal spindle-like, microcephaly associated) in human malignant gliomas Oncol Rep 2008 20 301 308 18636190

[B81] Riparbelli MG Callaini G Glover DM Avides Mdo C A requirement for the Abnormal Spindle protein to organise microtubules of the central spindle for cytokinesis in Drosophila J Cell Sci 2002 15 913 922 10.1242/jcs.115.5.913 11870210

[B82] do Carmo Avides M Glover DM Abnormal spindle protein, and the integrity of mitotic centrosomal microtubule organizing centers Science 1999 283 1733 1735 10.1126/science.283.5408.1733 10073938

[B83] Gonzalez C Saunders RD Casal J Molina I Carmena M Ripoll P Glover DM Mutations at the asp locus of Drosophila lead to multiple free centrosomes in syncytial embryos, but restrict centrosome duplication in larval neuroblasts J Cell Sci 1990 96 605 616 2283359 10.1242/jcs.96.4.605

[B84] Pulvers JN Bryk J Fish JL Wilsch-Bräuninger M Arai Y Schreier D Naumann R Helppi J Habermann B Vogt J Nitsch R Tóth A Enard W Pääbo S Huttner WB From the Cover: Mutations in mouse Aspm (abnormal spindle-like microcephaly associated) cause not only microcephaly but also major defects in the germline Proc Natl Acad Sci USA 2010 107 16595 16600 10.1073/pnas.1010494107 20823249 PMC2944708

[B85] Hung LY Tang CJ Tang TK Protein 4.1 R-135 interacts with a novel centrosomal protein (CPAP) which is associated with the gamma-tubulin complex Mol Cell Biol 2000 20 7813 7825 10.1128/MCB.20.20.7813-7825.2000 11003675 PMC86375

[B86] Hung LY Chen HL Chang CW Li BR Tang TK Identification of a novel microtubule-destabilizing motif in CPAP that binds to tubulin heterodimers and inhibits microtubule assembly Mol Biol Cell 2004 15 2697 2706 10.1091/mbc.E04-02-0121 15047868 PMC420094

[B87] Chen CY Olayioye MA Lindeman GJ Tang TK CPAP interacts with 14-3-3 in a cell cycle-dependent manner Biochem Biophys Res Commun 2006 342 1203 1210 10.1016/j.bbrc.2006.02.089 16516142

[B88] Gul A Hassan MJ Hussain S Raza SI Chishti MS Ahmad W A novel deletion mutation in *CENPJ *gene in a Pakistani family with autosomal recessive primary microcephaly J Hum Genet 2006 51 760 764 10.1007/s10038-006-0017-1 16900296

[B89] Al-Dosari MS Shaheen R Colak D Alkuraya FS Novel *CENPJ *mutation causes Seckel syndrome J Med Genet 2010 47 411 414 10.1136/jmg.2009.076646 20522431

[B90] Cho JH Chang CJ Chen CY Tang TK Depletion of CPAP by RNAi disrupts centrosome integrity and induces multipolar spindles Biochem Biophys Res Commun 2006 339 742 747 10.1016/j.bbrc.2005.11.074 16316625

[B91] Koyanagi M Hijikata M Watashi K Masui O Shimotohno K Centrosomal P4.1-associated protein is a new member of transcriptional coactivators for nuclear factor-kappaB J Biol Chem 280 12430 12437 10.1074/jbc.M410420200 15687488

[B92] Basto R Lau J Vinogradova T Gardiol A Woods CG Khodjakov A Raff JW Flies without centrioles Cell 2006 125 1375 1386 10.1016/j.cell.2006.05.025 16814722

[B93] Stevens NR Raposo AA Basto R St Johnston D Raff JW From stem cell to embryo without centrioles Curr Biol 2007 17 1498 1503 10.1016/j.cub.2007.07.060 17716897 PMC1971134

[B94] Kirkham M Müller-Reichert T Oegema K Grill S Hyman AA SAS-4 is a C. elegans centriolar protein that controls centrosome size Cell 2003 112 575 587 10.1016/S0092-8674(03)00117-X 12600319

[B95] Dammermann A Maddox PS Desai A Oegema K SAS-4 is recruited to a dynamic structure in newly forming centrioles that is stabilized by the gamma-tubulin-mediated addition of centriolar microtubules J Cell Biol 2008 180 771 785 10.1083/jcb.200709102 18299348 PMC2265562

[B96] Karkera JD Izraeli S Roessler E Dutra A Kirsch I Muenke M The genomic structure, chromosomal localization, and analysis of SIL as a candidate gene for holoprosencephaly Cytogenet Genome Res 2002 97 62 67 10.1159/000064057 12438740

[B97] Aplan PD Lombardi DP Ginsberg AM Cossman J Bertness VL Kirsch IR Disruption of the human SCL locus by "illegitimate" V-(D)-J recombinase activity Science 1990 250 1426142 1426149 10.1126/science.2255914 2255914

[B98] Pfaff KL Straub CT Chiang K Bear DM Zhou Y Zon LI The zebra fish cassiopeia mutant reveals that SIL is required for mitotic spindle organization Mol Cell Biol 2007 27 5887 5897 10.1128/MCB.00175-07 17576815 PMC1952118

[B99] Campaner S Kaldis P Izraeli S Kirsch IR Sil phosphorylation in a Pin1 binding domain affects the duration of the spindle checkpoint Mol Cell Biol 2005 25 6660 6672 10.1128/MCB.25.15.6660-6672.2005 16024801 PMC1190358

[B100] Izraeli S Lowe LA Bertness VL Good DJ Dorward DW Kirsch IR Kuehn MR The SIL gene is required for mouse embryonic axial development and left-right specification Nature 1999 399 691 694 10.1038/21429 10385121

[B101] Qamar R Ayub Q Mohyuddin A Helgason A Mazhar K Mansoor A Zerjal T Tyler-Smith C Mehdi SQ Y-chromosomal DNA variation in Pakistan Am J Hum Genet 2002 70 1107 1124 10.1086/339929 11898125 PMC447589

[B102] Mehdi SQ Qamar R Ayub Q Kaliq S Mansoor A Ismail M Hammer MF Underhill PA Cavalli-Sforza LL Papiha SS, Deka R, Chakraborty R The origins of Pakistani populations: evidence from Y chromosome markers Genomic diversity: applications in human population genetics 2002 New York Kluwer Academic/Plenum Publishers 83 90

[B103] Cavalli-Sforza LL Piazza A Human genomic diversity in Europe: a summary of recent research and prospects for the future Eur J Hum Genet 1993 1 3 18 7520820 10.1159/000472383

[B104] Firasat S Khaliq S Mohyuddin A Papaioannou M Tyler-Smith C Underhill PA Ayub Q Y-chromosomal evidence for a limited Greek contribution to the Pathan population of Pakistan Eur J Hum Genet 2007 15 121 126 10.1038/sj.ejhg.5201726 17047675 PMC2588664

[B105] Kousar R Nawaz H Khurshid M Ali G Khan SU Mir H Ayub M Wali A Ali N Jelani M Basit S Ahmad W Ansar M Mutation analysis of the *ASPM *gene in 18 Pakistani families with autosomal recessive primary microcephaly J Child Neurol 2010 25 715 720 10.1177/0883073809346850 19808985

[B106] Dorus S Vallender EJ Evans PD Anderson JR Gilbert SL Meadowland M Wyckoff GJ Malcom CM Lahn BT Accelerated evolution of nervous system genes in the origin of Homo sapiens Cell 2004 119 1027 1040 10.1016/j.cell.2004.11.040 15620360

[B107] Evans PD Anderson JR Vallender EJ Gilbert SL Malcom CM Dorus S Lahn BT Adaptive evolution of *ASPM*, a major determinant of cerebral cortical size in humans Hum Mol Genet 2004 13 489 494 10.1093/hmg/ddh055 14722158

[B108] Evans PD Anderson JR Vallender EJ Choi SS Lahn BT Reconstructing the evolutionary history of microcephalin, a gene controlling human brain size Hum Mol Genet 2004 13 1139 1145 10.1093/hmg/ddh126 15056607

[B109] Evans PD Gilbert SL Mekel-Bobrov N Vallender EJ Anderson JR Vaez-Azizi LM Tishkoff SA Hudson RR Lahn BT Microcephalin, a gene regulating brain size, continues to evolve adaptively in humans Science 2005 309 1717 1720 10.1126/science.1113722 16151009

[B110] Mekel-Bobrov N Gilbert SL Evans PD Vallender EJ Anderson JR Hudson RR Tishkoff SA Lahn BT Ongoing adaptive evolution of *ASPM*, a brain size determinant in Homo sapiens Science 2005 309 1720 1722 10.1126/science.1116815 16151010

[B111] Currat M Excoffier L Maddison W Otto SP Ray N Whitlock MC Yeaman S Comment on "Ongoing adaptive evolution of ASPM, a brain size determinant in Homo sapiens" and "Microcephalin, a gene regulating brain size, continues to evolve adaptively in humans" Science 2006 313 172 179 16840683 10.1126/science.1122822

[B112] Mekel-Bobrov N Posthuma D Gilbert SL Lind P Gosso MF Luciano M Harris SE Bates TC Polderman TJ Whalley LJ Fox H Starr JM Evans PD Montgomery GW Fernandes C Heutink P Martin NG Boomsma DI Deary IJ Wright MJ de Geus EJ Lahn BT The ongoing adaptive evolution of ASPM and Microcephalin is not explained by increased intelligence Hum Mol Genet 2007 16 600 608 17220170 10.1093/hmg/ddl487

[B113] Rushton JP Vernon PA Bons TA No evidence that polymorphisms of brain regulator genes *Microcephalin *and *ASPM *are associated with general mental ability, head circumference or altruism Biol Lett 2007 3 157 160 10.1098/rsbl.2006.0586 17251122 PMC2104484

[B114] Yu F Hill RS Schaffner SF Sabeti PC Wang ET Mignault AA Ferland RJ Moyzis RK Walsh CA Reich D Comment on "Ongoing adaptive evolution of ASPM, a brain size determinant in Homo sapiens" Science 2007 316 370 375 17446375 10.1126/science.316.5823.370a

[B115] Wang JK Li Y Su B A common SNP of MCPH1 is associated with cranial volume variation in Chinese population Hum Mol Genet 2008 17 1329 1335 10.1093/hmg/ddn021 18204051

[B116] Uniprot protein website http://www.uniprot.org/uniprot/?query=domain:%22IQ+domain*%22

[B117] Woods RP Freimer NB De Young JA Fears SC Sicotte NL Service SK Valentino DJ Toga AW Mazziotta JC Normal variants of Microcephalin and ASPM do not account for brain size variability Hum Mol Genet 2006 15 2025 2029 10.1093/hmg/ddl126 16687438

[B118] Rivero O Sanjuán J Moltó MD Aguilar EJ Gonzalez JC de Frutos R Nájera C The microcephaly *ASPM *gene and schizophrenia: A preliminary study Schizophr Res 2006 84 427 429 10.1016/j.schres.2006.03.009 16631353

[B119] Dediu D Ladd DR Linguistic tone is related to the population frequency of the adaptive haplogroups of two brain size genes, *ASPM *and *Microcephalin* Proc Natl Acad Sci USA 2007 104 10944 109449 10.1073/pnas.0610848104 17537923 PMC1904158

[B120] Montgomery SH Capellini I Venditti C Barton RA Mundy NI Adaptive evolution of four microcephaly genes and the evolution of brain size in anthropoid primates Mol Biol Evol 2011 28 625 638 10.1093/molbev/msq237 20961963

